# Influence of the migration of radioactive contaminants in soil, resident occupancy, and variability in contamination on isodose lines for typical Northern European houses

**DOI:** 10.1038/s41598-019-44392-z

**Published:** 2019-05-27

**Authors:** Yvonne Hinrichsen, Robert Finck, Johan Martinsson, Christopher Rääf, Kasper Grann Andersson

**Affiliations:** 1 Technical University of Denmark, Center for Nuclear Technologies, Roskilde, 4000 Denmark; 2 Lund University, Department of Translational Medicine, Medical Radiation Physics, Malmö, 205 02 Sweden

**Keywords:** Software, Computational science

## Abstract

In the remedial phase following an accidental radioactive release, it is important that soil decontamination measures are carried out on the areas that contribute most to the radiation dose. In this study, the newly developed concept of isodose lines was applied to the area around typical Swedish dwellings to identify these areas. The influence of the most common building materials in Sweden, wood and brick, and the importance of the positions of doors and windows on the isodose lines were demonstrated for specific positions inside the houses, as well as for the entire house, assuming the residents exhibit typical resident occupancy. Decontamination of the areas within certain isodose lines was shown to result in a greater dose reduction than decontaminating the same area of soil within a certain distance of the house. Furthermore, the impact of vertical migration of the radioactive contaminants in the soil on the isodose lines was studied, showing that the area enclosed by isodose lines decreases over time as the contaminants migrate deeper into the soil. The resulting isodose lines and their change over time are dominated by the downward movement of the contamination in the upper layer of soil. The impact of the variability in contamination on the final isodose lines and their dependence on building materials are demonstrated.

## Introduction

The external radiation exposure is an important contribution to the radiation exposure of the population after the release of gamma-ray-emitting radionuclides into the air and their deposition on the ground and on other structures^1^. In inhabited environments, this radiation exposure can be reduced by building structures, depending on the geometry of the buildings, the deposition distribution on the different surfaces, and resident occupancy. The removal of a thin layer of soil on unpaved areas can reduce the contamination by as much as 90%, provided the depth is optimized according to the vertical distribution of the contaminants^2^. The cost of employing skilled personnel, as well as equipment and consumables for such measures can be very high, and the construction of complex waste repositories may also be necessary^3^. It is therefore important to concentrate decontamination activities on the areas that have the greatest impact on the radiation exposure of the population, taking into account the reduction in exposure provided by various buildings.

The isodose concept was recently developed for this purpose^4^. When considering the dose contribution resulting from radionuclides deposited on the ground, this concept illustrates the extent to which the surrounding areas contribute to the external radiation exposure at different representative observation points inside a building, and can thus be used to optimize topsoil removal following a radioactive release. The aim of this study was to apply the isodose concept to two typical Swedish residential houses, constructed different materials, and to determine isodose lines at various locations in the houses, as well as for the entire the houses by applying data on typical resident occupancy. The impact of vertical migration of the contaminants in the soil and variability in the contaminants on the isodose lines was also studied.

## Results

### Monte Carlo computed isodose lines around typical Swedish houses

**Figure 1:**
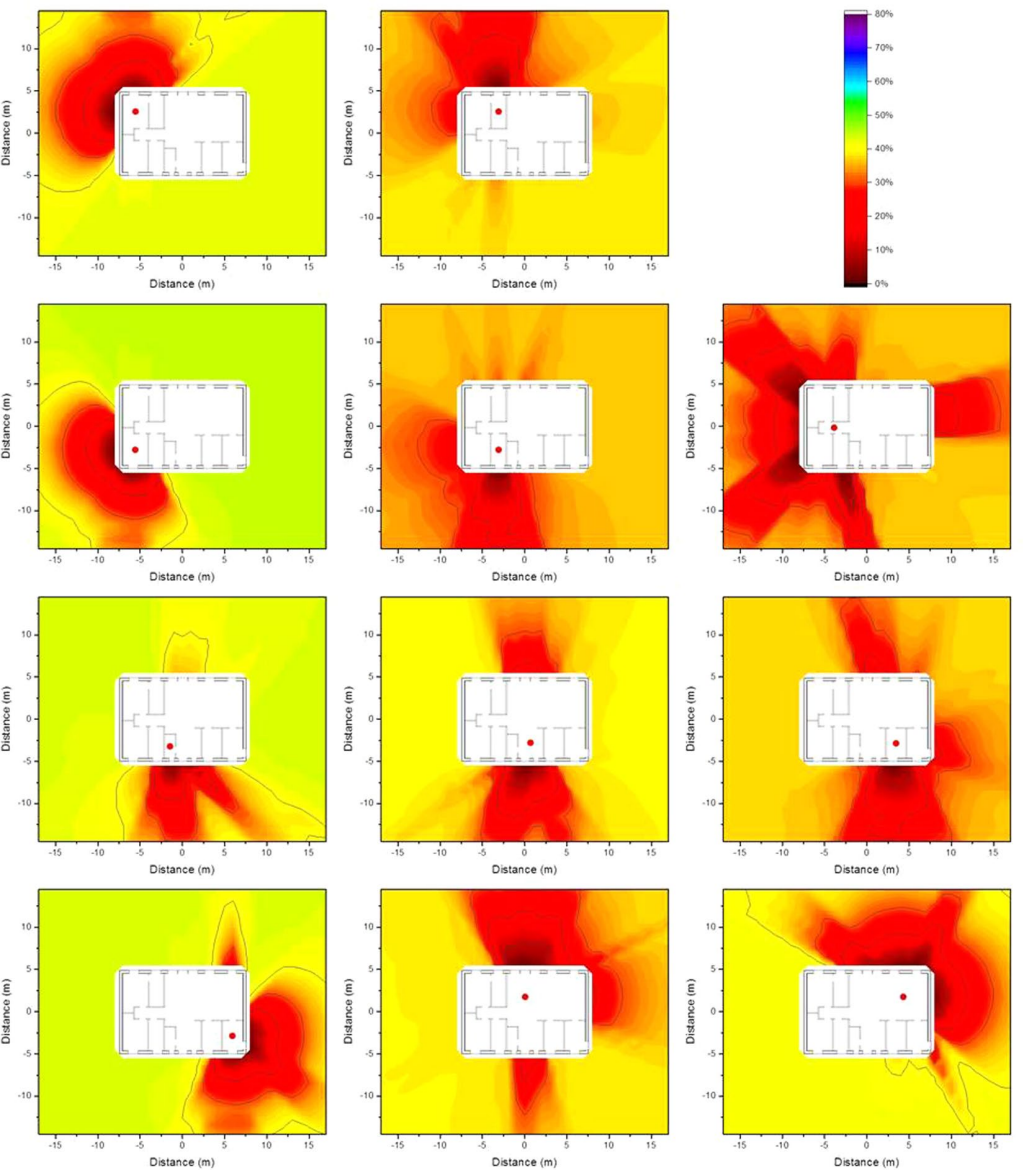
Isodose lines around the wooden house at the eleven observation points defined above (red dots) resulting from homogeneous ^137^Cs contamination at ground level. The shading indicates the fraction of dose contribution to the observation point including the areas that are surrounded by the respective one. When the outside line for the isodose line of a certain relative dose reduction reaches the limit of the calculation grid, its shape may differ for a larger calculation grid.

**Figure 2:**
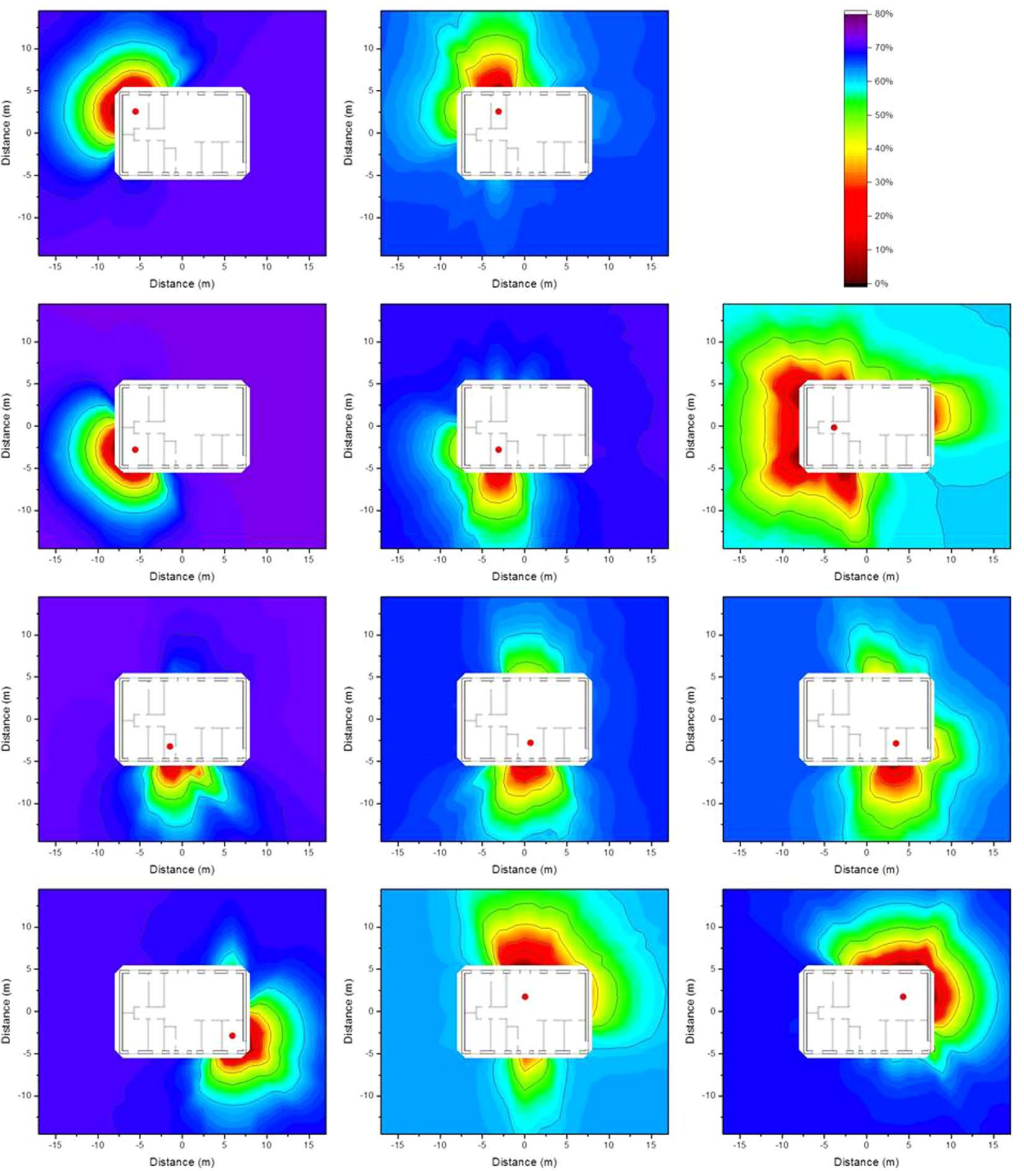
Isodose lines around the wooden house at the eleven observation points defined above (red dots) resulting from homogeneous ^137^Cs contamination 2.5 cm below ground level. The shading indicates the fraction of dose contribution to the observation point including the areas that are surrounded by the respective one. When the outside line for the isodose line of a certain relative dose reduction reaches the limit of the calculation grid, its shape may differ for a larger calculation grid.

**Figure 3:**
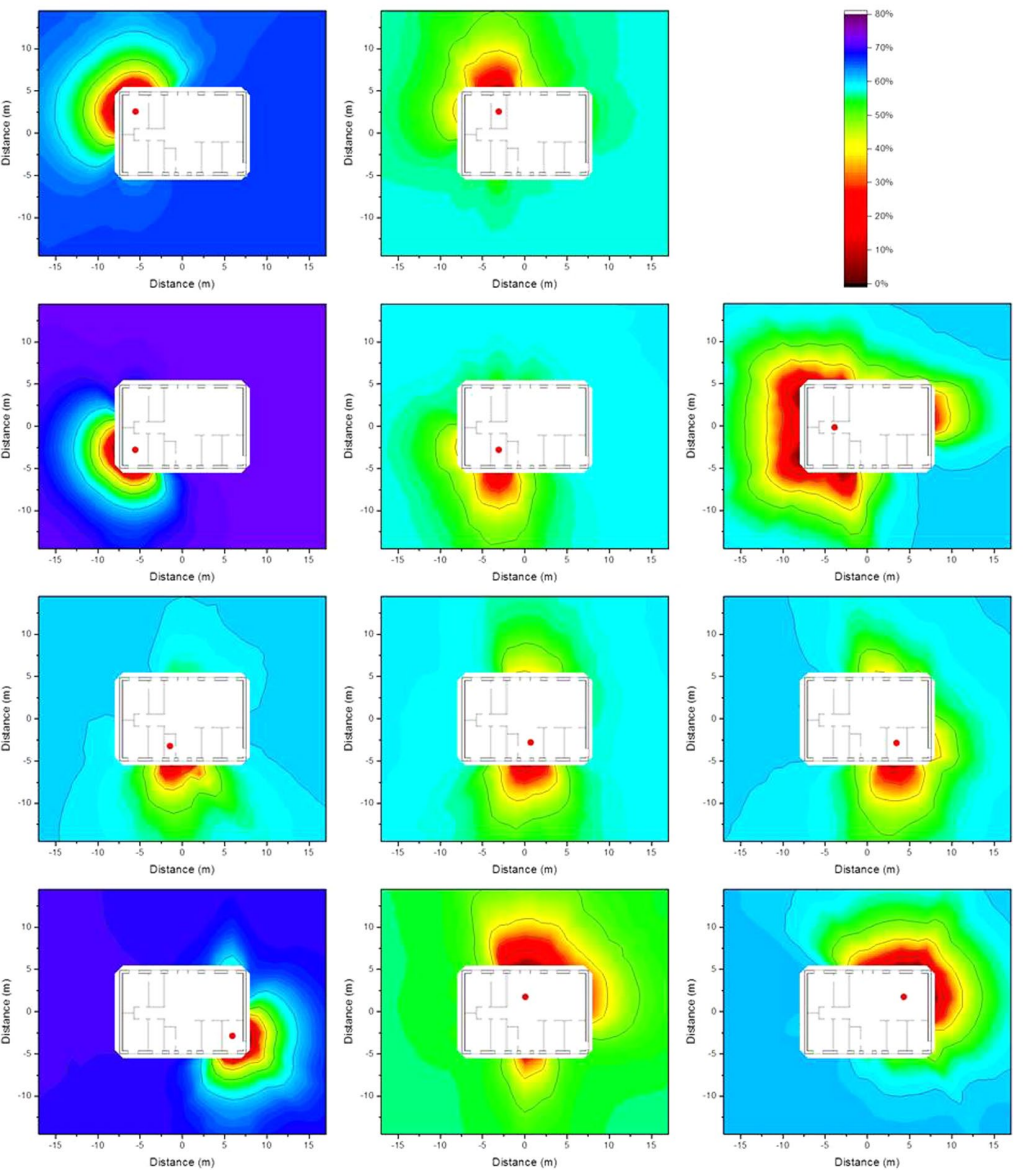
Isodose lines around the wooden house at the eleven observation points defined above (red dots) resulting from homogeneous ^137^Cs contamination 5 cm below ground level. The shading indicates the fraction of dose contribution to the observation point including the areas that are surrounded by the respective one. When the outside line for the isodose line of a certain relative dose reduction reaches the limit of the calculation grid, its shape may differ for a larger calculation grid.

**Figure 4:**
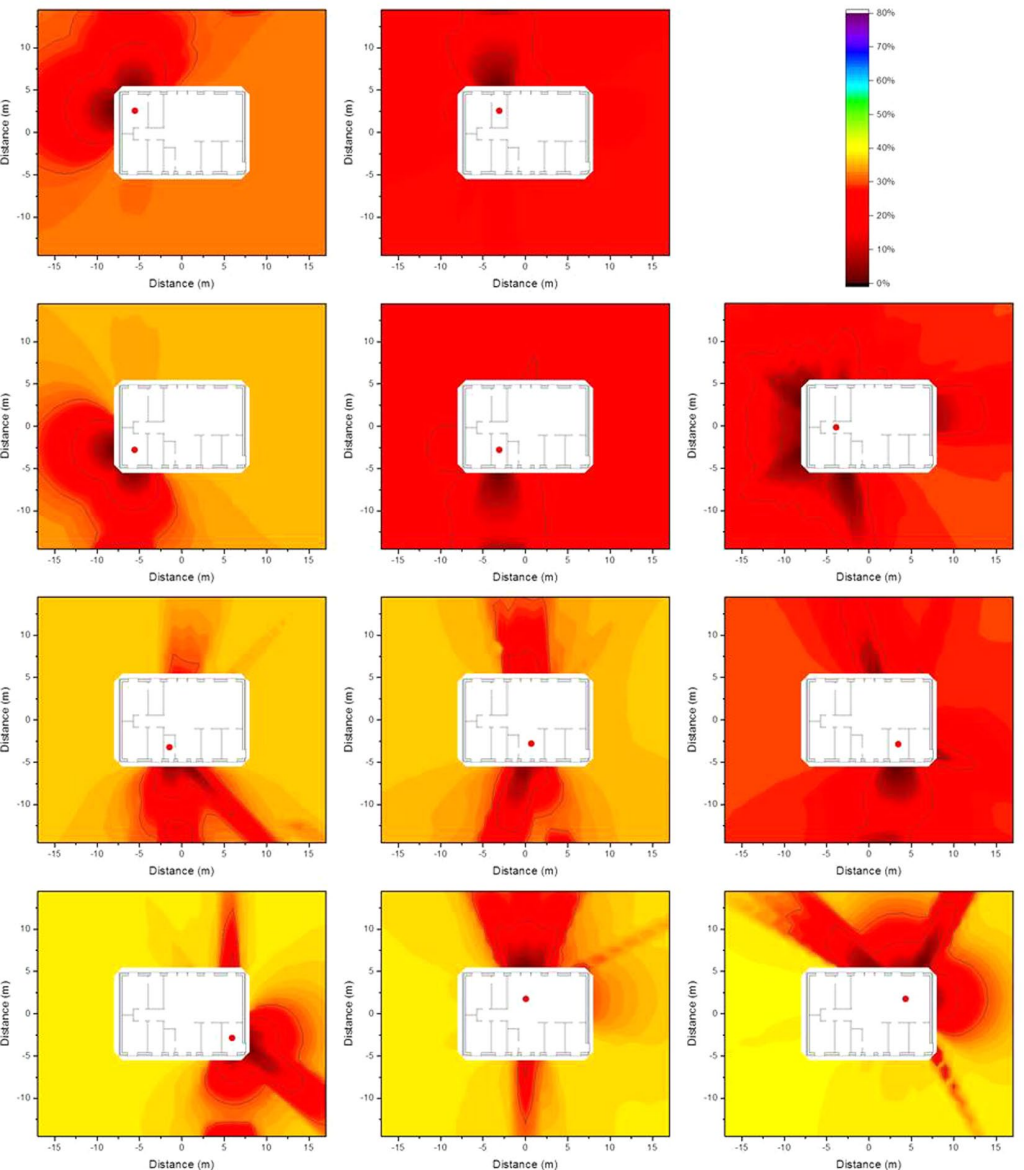
Isodose lines around the brick house at the eleven observation points defined above (red dots) resulting from homogeneous ^137^Cs contamination at ground level. The shading indicates the fraction of dose contribution to the observation point including the areas that are surrounded by the respective one. When the outside line for the isodose line of a certain relative dose reduction reaches the limit of the calculation grid, its shape may differ for a larger calculation grid.

**Figure 5:**
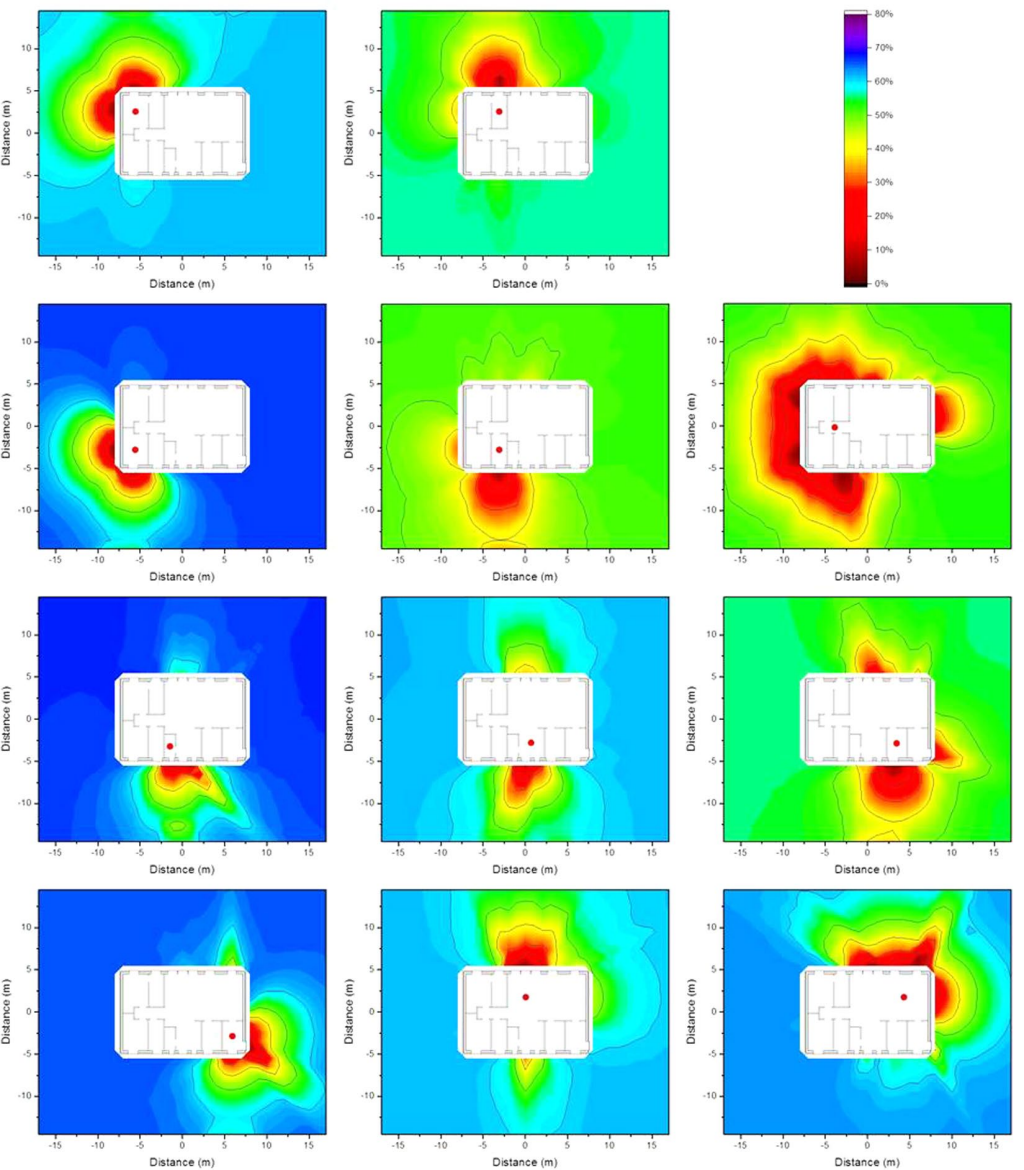
Isodose lines around the brick house at the eleven observation points defined above (red dots) resulting from homogeneous ^137^Cs contamination 2.5 cm below ground level. The shading indicates the fraction of dose contribution to the observation point including the areas that are surrounded by the respective one. When the outside line for the isodose line of a certain relative dose reduction reaches the limit of the calculation grid, its shape may differ for a larger calculation grid.

**Figure 6:**
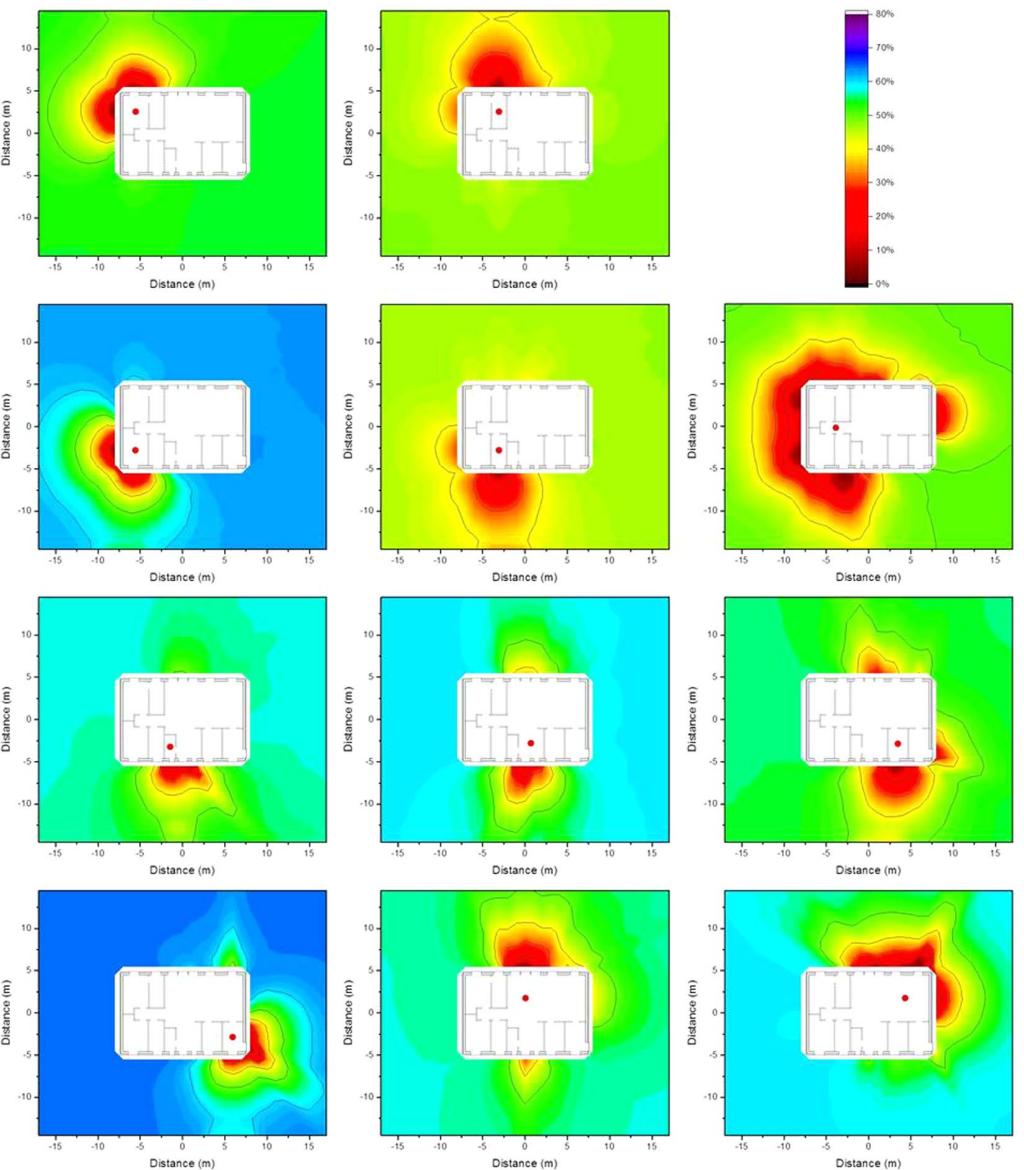
Isodose lines around the brick house at the eleven observation points defined above (red dots) resulting from homogeneous ^137^Cs contamination 5 cm below ground level. The shading indicates the fraction of dose contribution to the observation point including the areas that are surrounded by the respective one. When the outside line for the isodose line of a certain relative dose reduction reaches the limit of the calculation grid, its shape may differ for a larger calculation grid.

The isodose concept as described in Equation 1 in the Method section was applied to a case in which decontamination would lead to different reductions in the absorbed dose, depending on which of the eleven different observation points is considered. Homogeneous ^137^Cs contamination was assumed on the ground surrounding the wooden and the brick houses, as well as 2.5 cm and 5 cm below ground level. The results are presented graphically as isodose lines in Figs 1, 2 and 3 for the wooden house and in Figs 4, 5 and 6 for the brick house.

It can be seen from the Figures above that the shape of the areas encompassed by the isodose lines for a given observation point are relatively similar for all depths of the contamination, and for both the wooden and brick houses. The shapes of the isodose lines reflect the different materials, as well as the positions of doors and windows as they shield less than the walls. The zones for deposited contamination below ground level are smaller as fewer gamma photons from remote areas reach the observation point, as they lose energy through interactions with the soil. To obtain a better estimate of the size of the area that would have to be decontaminated to achieve a certain relative dose reduction for contamination at different depths, values of the primary dose factor were calculated for an infinite contaminated surface, and are presented in Table 1. The primary dose factor is directly related to the dose to the residents when no decontamination measures are implemented, and is expressed in pGy per γmm^−2^, representing the dose (pGy) that would result from a homogeneous plane source at ground level, and at 2.5 cm and 5 cm below ground level, for a source with a strength of one gamma photon per unit area (γmm^−2^). When determining isodose lines, it represents the total dose at the observation point *i*, *D*_*i*,∞_, in Equation 1 described in the Method section. It should be borne in mind that the isodose lines illustrate relative dose reduction and not the actual dose reduction.

**Table 1:** Primary dose factors before decontamination (pGy per γmm^−2^) for the eleven observation points inside the wooden house and the brick house resulting from contamination at ground level, and 2.5 cm and 5 cm below ground level.

Observation point	Wooden house			Brick house		
	Contamination depth			Contamination depth		
	0 cm	2.5 cm	5 cm	0 cm	2.5 cm	5 cm
1	216	42.4	23.5	94.0	16.8	10.1
2	99.0	17.2	9.87	54.0	8.60	5.16
3	201	40.9	21.4	85.0	15.2	8.54
4	93.1	15.1	9.07	54.9	8.45	5.03
5	84.1	12.3	6.04	34.6	5.55	3.04
6	108	20.4	12.5	66.5	11.0	6.73
7	156	26.7	15.4	112	19.1	10.1
8	132	21.9	12.1	80.5	11.6	6.05
9	246	51.6	27.0	134	26.1	13.9
10	183	29.3	16.1	131	22.3	11.6
11	208	34.2	18.6	110	18.3	9.76

The values of the primary dose factors at the different observation points vary by up to a factor of five. Furthermore, the primary dose factors for contamination at ground level are about 5–6 times higher than the respective factors for contamination 2.5 cm below ground level, and those about 2 times higher than the corresponding factors for contamination 5 cm below ground level. Moreover, the primary dose factors inside the wooden house are about twice those in the brick house.

### Isodose lines according to resident occupancy

**Figure 7:**
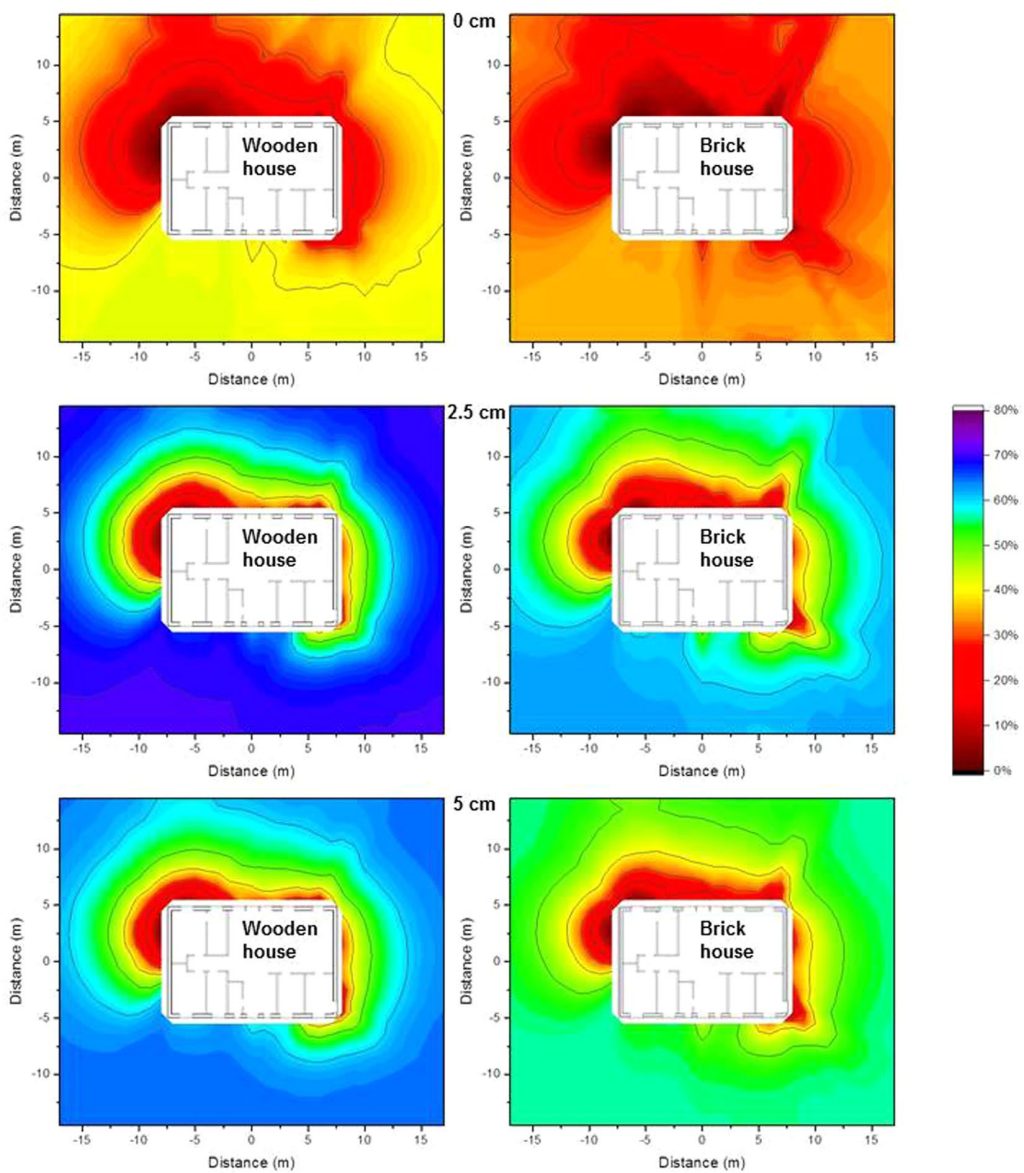
Isodose lines around the wooden house (left) and the brick house (right) using typical resident occupancy factors resulting from homogeneous ^137^Cs contamination at ground level (top), 2.5 cm below (middle), and 5 cm below ground level (bottom). The shading indicates the fraction of dose contribution to the observation point including the areas that are surrounded by the respective one. When the outside line for a certain dose reduction reaches the limit of the calculation grid, its shape might differ for a larger calculation grid.

The occupancy factors, *p*_*i*_, as described in Equation 2 in the Method section were applied to determine isodose lines that are more representative of the dose to which a resident is exposed inside the house. The occupancy factors were chosen based the data published in the European EXPOLIS project^5–7^), in which thousands of people in seven European cities (Athens, Basel, Grenoble, Helsinki, Milan, Oxford, and Prague), were studied with respect to their time budgets, and the hours they spent in various microenvironments. From these data it was found that people spend about 14 h indoors at home, and about 1 of these 14 h preparing food in the kitchen. Further surveys show that people spend about 1 h eating^8^ (i.e. in the dining room), about 8 h sleeping^9^ (i.e. in the bedroom), and about 0.5 h in the bathroom^10^, leaving about 3.5 h which it is assumed is spent in the living room. The resulting isodose lines taking into account the occupancy factors determined by the named occupancy times are presented graphically in Fig. 7.

The isodose lines in Fig. 7 show the combination of the influence of the time spend in one room (e.g. bedroom) and continuous influence of building materials (e.g. door and window in the kitchen). It can be seen that the isodose lines for a wooden house are gentler than those for a brick house, as timber provides less shielding, and therefore has less impact on the isodose lines. Furthermore, the corresponding zones for the brick house are larger than those for the wooden house. The size of the zone 2.5 cm below ground level is only half of that at ground level. However, at a depth of 5 cm the zones appear to increase slightly, as the contribution from areas far away becomes insignificant.

### Impact of vertical migration of contaminants in the soil on the isodose lines

**Figure 8:**
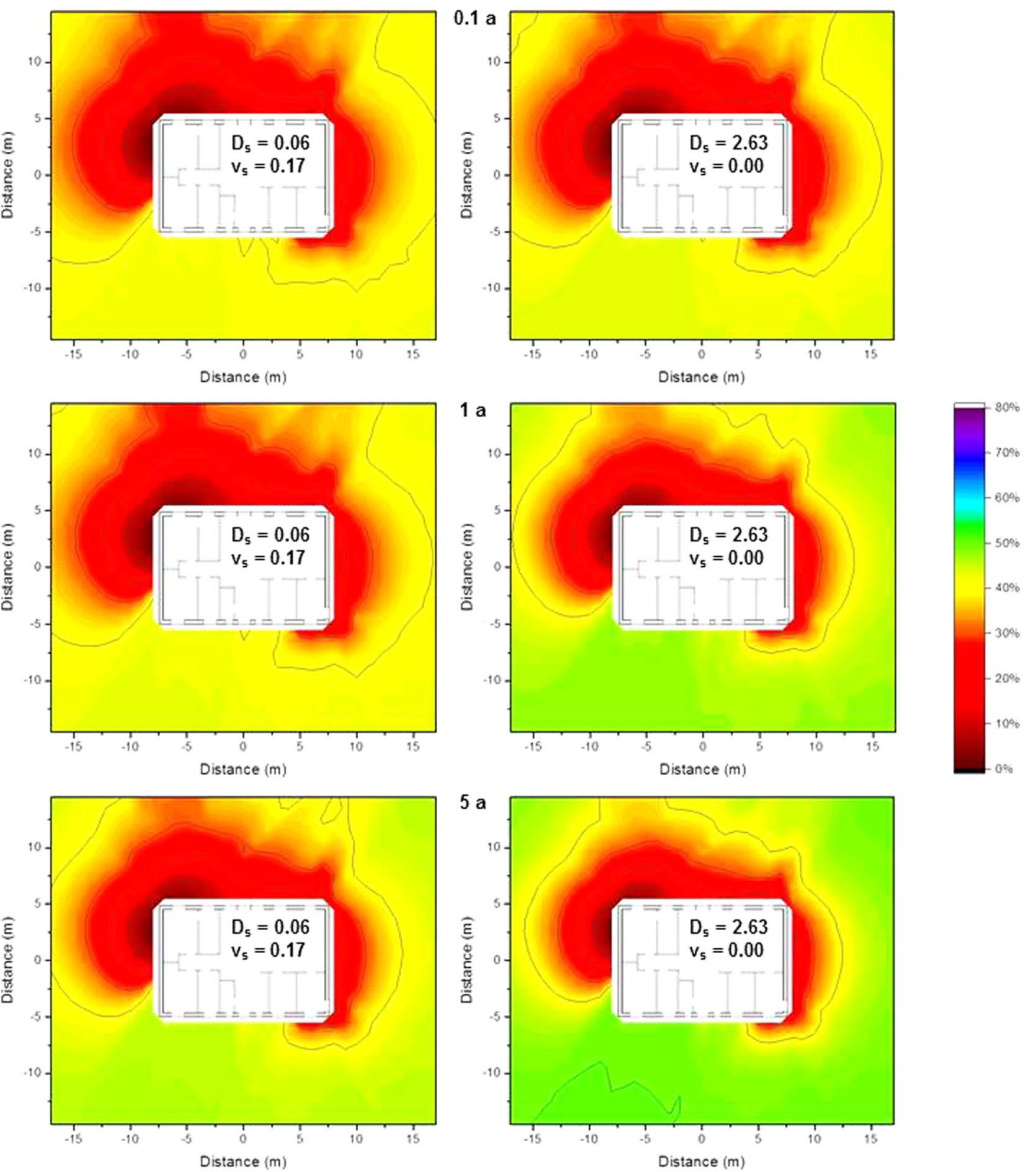
Isodose lines around the wooden house using typical resident occupancy factors resulting from homogeneous ^137^Cs contamination for a vertical distribution, 0.1 years (top), 1 year (middle), and 5 years after deposition (bottom), based on the two most extreme parameter combinations determined for western Sweden^11^. The shading indicates the fraction of dose contribution at the observation point including the areas that are surrounded by the respective one. When the outside line for a certain dose reduction reaches the limit of the calculation grid, its shape might differ for a larger calculation grid.

**Figure 9:**
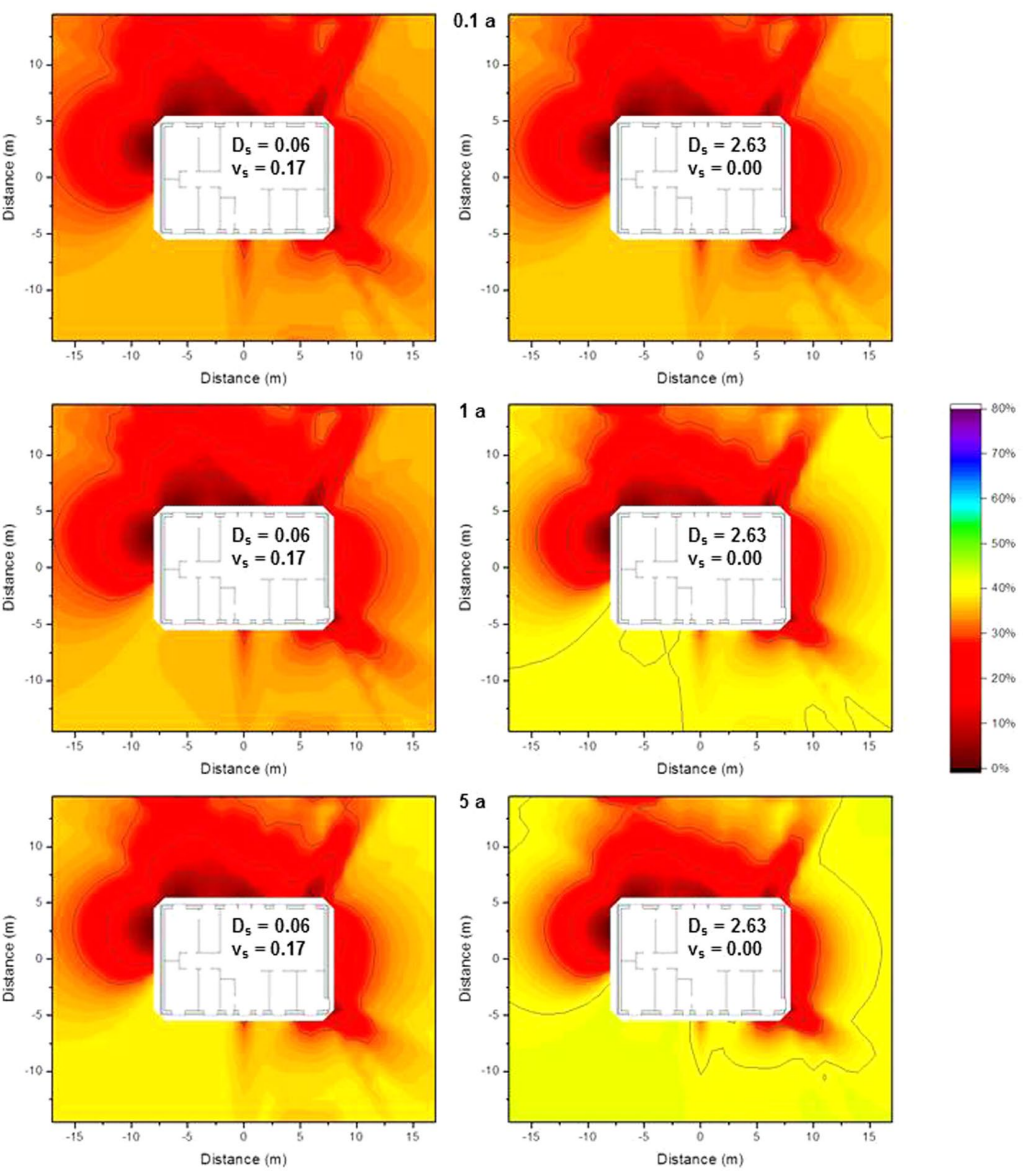
Isodose lines around the brick house using typical resident occupancy factors resulting from homogeneous ^137^Cs contamination for a vertical distribution, 0.1 years (top), 1 year (middle), and 5 years after deposition (bottom), based on the two most extreme parameter combinations determined for western Sweden^11^. The shading indicates the fraction of dose contribution at the observation point including the areas that are surrounded by the respective one. When the outside line for a certain dose reduction reaches the limit of the calculation grid, its shape might differ for a larger calculation grid.

As contaminants migrate downwards in the ground over time, the impact of the depth of the contamination on the isodose lines is of interest. The most extreme combinations of the values of the effective dispersion coefficient *D*_*s*_ and the convective velocity *v*_*s*_ (Equation 3 in the Method section) determined for ^137^Cs by Almgren and Isaksson^11^ for sampling sites in western Sweden (*D*_*s*_ = 0.06 cm^2^ a^−1^, *v*_*s*_ = 0.17 cm a^−1^ and *D*_*s*_ = 2.63 cm^2^ a^−1^ and *v*_*s*_ = 0.00 cm a^−1^) were chosen. The contaminant distributions were calculated for the first 5 cm of soil for times of 0.1 a, 1 a, and 5 a after deposition and multiplied by the air kerma free-in-air values interpolated from the calculations at ground level, and depths of 2.5 cm and 5 cm. The results are presented graphically as isodose lines for the wooden house in Fig. 8 and for the brick house in Fig. 9.

From Figs 8 and 9 it can be seen that the differences in the isodose lines for the wooden and brick houses are similar to those observed in the Fig. 7. It can also be seen that for these parameter combinations the isodose lines are similar on the short timescale of 0.1 a. Over longer timescales the respective zones become smaller as the contaminants migrate deeper into the soil. This effect is stronger for the parameter combination of *D*_*s*_ = 2.63 cm^2^ a^−1^ and *v*_*s*_ = 0.00 cm a^−1^ than for *D*_*s*_ = 0.06 cm^2^ a^−1^, *v*_*s*_ = 0.17 cm a^−1^. Moreover, it can be seen that the upper layer of soil dominates as it contributes more to the air kerma free-in-air than the lower soil layer of soil, as indicated by the primary dose factors presented in Table 1.

In the comparison of the wooden and the brick house similar differences can be seen as described before. The parameter combination shows that the isodose lines are similar for a short timescale like 0.1 a. Over longer timescales the respective zones become smaller as the contaminants migrate to deeper soil level. This effect is stronger for the parameter combination *D*_*s*_ = 2.63 cm^2^ a^−1^ and *v*_*s*_ = 0.00 cm a^−1^ than for *D*_*s*_ = 0.06 cm^2^ a^−1^, *v*_*s*_ = 0.17 cm a^−1^. Moreover, the dominance of the top soil layer can be seen as it contributes more to the air kerma free-in-air than the lower soil level as shown for the primary dose factor presented in Table 1.

### Impact of contamination variability on the isodose lines

**Figure 10:**
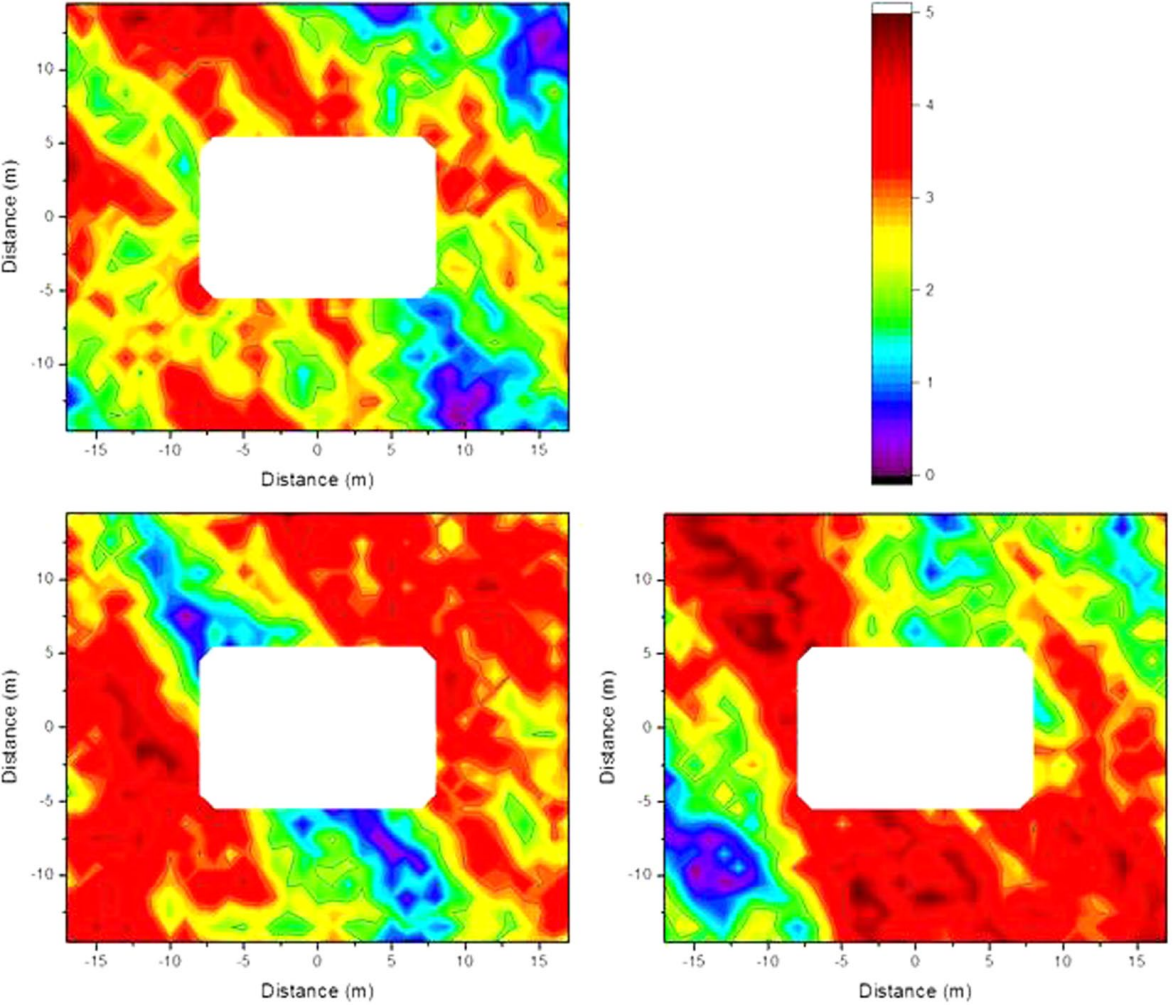
Three different variations in ^137^Cs contamination obtained using a random number generator.

**Figure 11:**
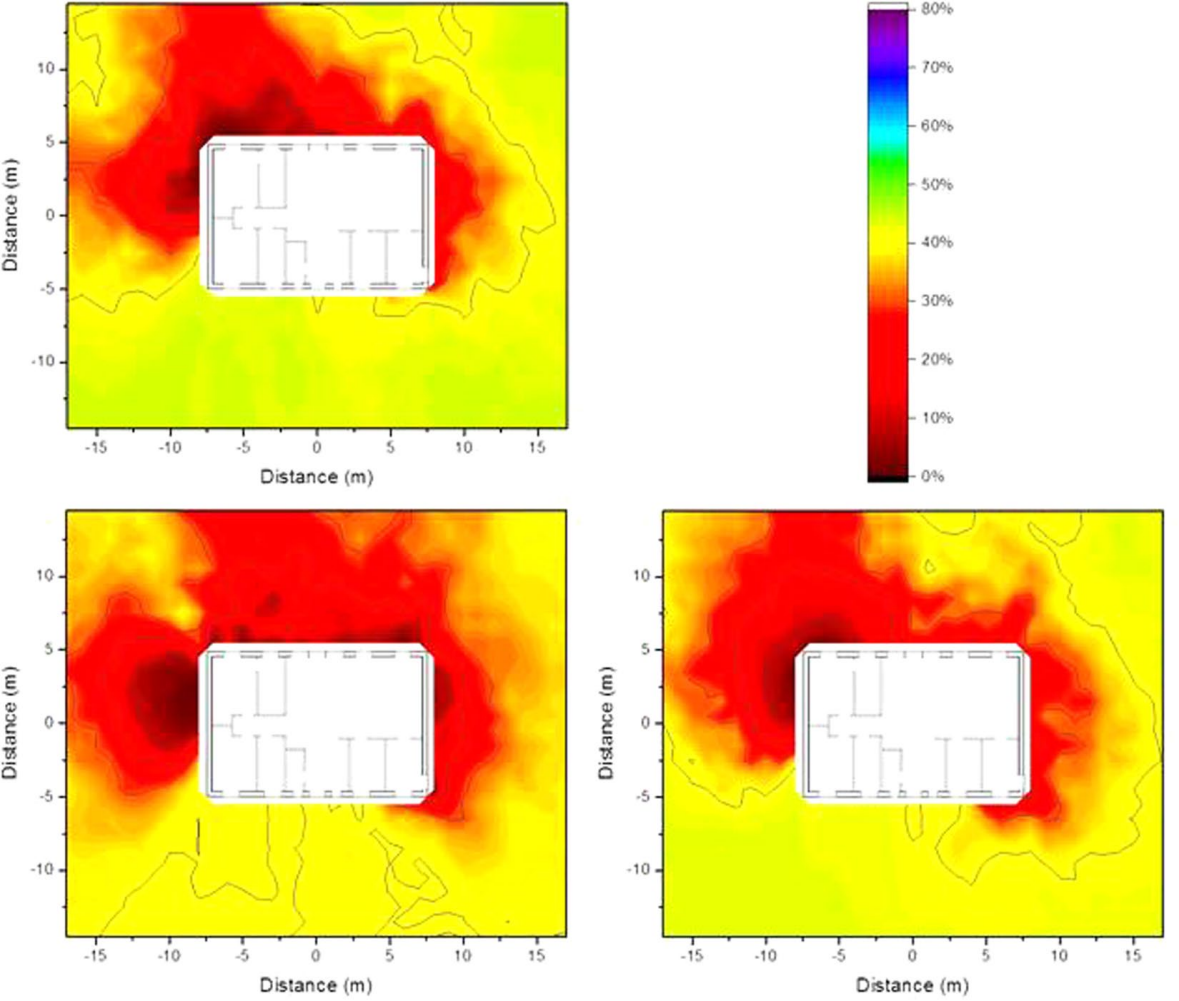
Isodose lines around the wooden house using typical resident occupancy factors for three different variability scenarios of ^137^Cs contamination, according to Fig. 10. The shading indicates the fraction of dose contribution to the observation point including the areas that are surrounded by the respective one. When the outside line for a certain dose reduction reaches the limit of the calculation grid, its shape might differ for a larger calculation grid.

**Figure 12:**
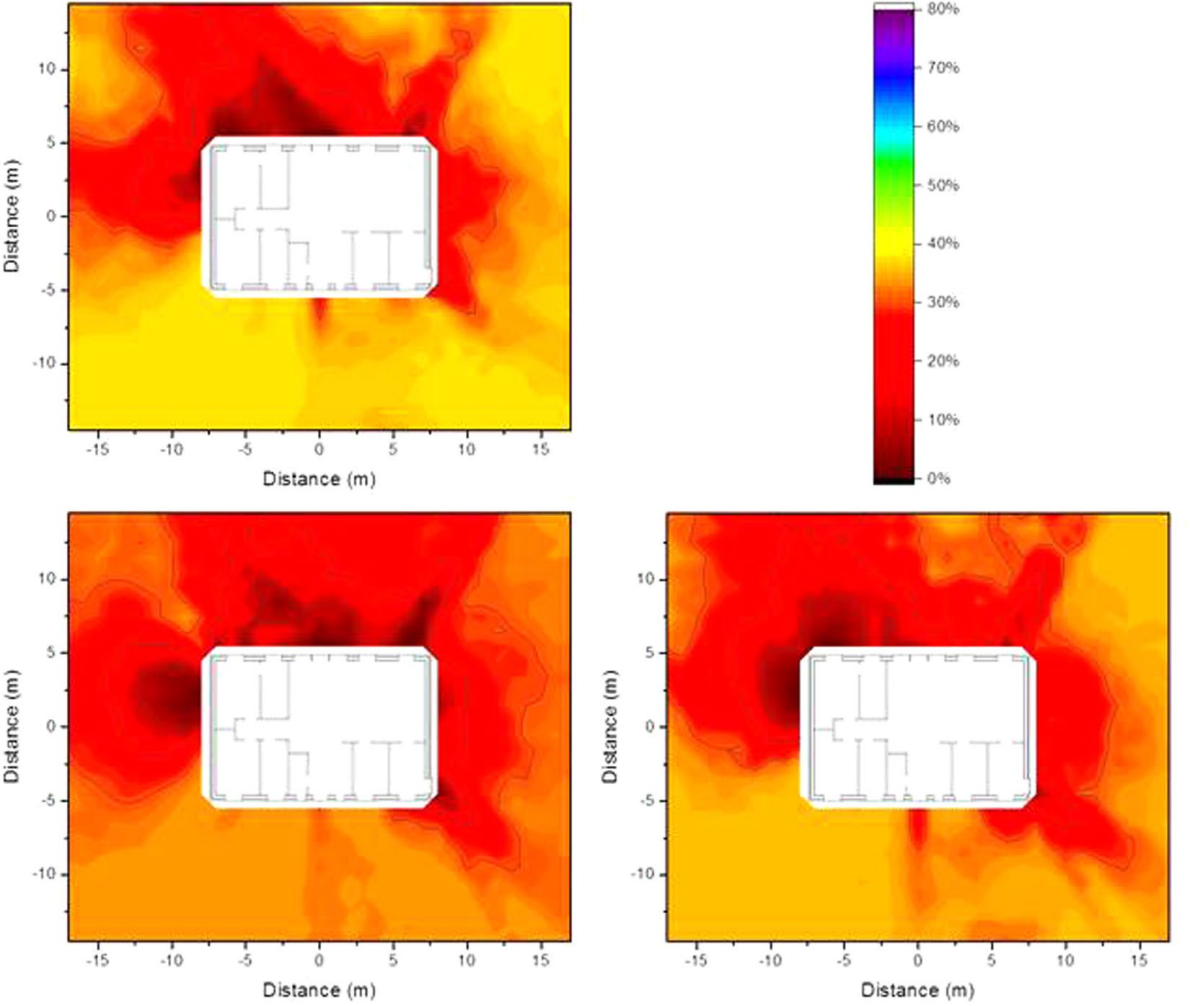
Isodose lines around the brick house using typical resident occupancy factors for three different variability scenarios of ^137^Cs contamination, according to Fig. 10. The shading indicates the fraction of dose contribution to the observation point including the areas that are surrounded by the respective one. When the outside line for a certain dose reduction reaches the limit of the calculation grid, its shape might differ for a larger calculation grid.

The model for contamination variability is based on measurements of ^137^Cs fallout in settlements in Russia and Belarus following the Chernobyl nuclear power plant accident^12^, where dose rate levels represented by ^137^Cs peak gamma signals ranging from 0 till 5 kcps were measured 0.1 m above a 9 m × 9 m open, untouched grass surface. A random number generator picking values from 0 till 5 was applied to the 1 m × 1 m grid that was used for the Monte Carlo calculations described in the Method section with the restriction that the values in all neighboring fields on a horizontal or vertical line are allowed to differ by at most ±1, and on a diagonal line by at most ±1.4. These values were applied as dimensionless scaling factors by multiplication of the Monte Carlo calculated air kerma free-in-air values for the respective fields that were determined for contamination at ground level (as this dominates the radiation dose to residents), for the wooden and the brick house. The primary dose factors were determined by subtracting the one for homogeneous contamination by the sum of the air kerma free-in-air values determined without the scaling factor, then multiplied by the average of the randomly generated multiplication factors and finally the sum of determined air kerma free-in-air values with multiplication factor was added. This was done for three different contamination variability scenarios obtained with the random number generator (Fig. 10). The resulting isodose lines are presented graphically in for the wooden house in Fig. 11 and for the brick house in Fig. 12.

Comparing the isodose lines for the three contamination variability scenarios with those determined for homogeneous contamination (First line in Fig. 7) shows that the original shape is still visible. However, as the shielding of the wooden house is lower, the effect of contamination variability on the isodose lines is greater than in the case of the brick house. This is supported by the values of the Pearson correlation coefficient, being 0.96, 0.93, and 0.95 for the wooden house and 0.96, 0.94, and 0.95 for the brick house, for the three different contamination variability scenarios.

To investigate the benefit of decontamination according to isodose lines that were determined for homogeneous contamination, in case of contamination variability, the dose factors were calculated after decontamination of an area up to 2 m from the houses (116 m^2^), as well as the respective dose factor after decontamination of 116 m^2^ according to the previously determined isodose lines. The values obtained are given in Table 2 for both types of houses. Relative dose reductions were calculated for both decontamination scenarios by dividing the dose factors decontamination by the respective primary dose factor. The relative dose reduction after optimized was compared to the relative dose reduction after normal decontamination by a ratio. The primary dose factors, the relative dose reduction, and the ratio comparing the relative dose reductions for optimized decontamination according to isodose lines and for normal decontamination within 2 m from the houses are also included in Table 2.

**Table 2:** Primary dose factors before decontamination (pGy per γmm^−2^) for homogeneous ^137^Cs contamination and three variability scenarios at ground level, using typical resident occupancy factors in the wooden and the brick house, together with the dose factors obtained after normal decontaminating an area of 116 m^2^ directly around the houses, or optimized decontamination of the same area but according to the isodose lines presented in the first line of Fig. 7, including relative dose reductions, and ratio of the relative dose reductions.

	Primary dose factor	After normal decontamination		After optimized decontamination		Ratio of the relative dose reductions
		Dose factor	Relative dose reduction	Dose factor	Relative dose reduction	
Wooden house:						
Homogeneous contamination	209	177	15.5%	165	21.2%	1.37
Variability scenario 1	217	184 6	15.3%	167	23.0%	1.50
Variability scenario 2	202	178	12.1%	168	17.1%	1.42
Variability scenario 3	213	178	16.3%	165	22.8%	1.39
Brick house:						
Homogeneous contamination	102	90	12.3%	84	18.4%	1.47
Variability scenario 1	106	93	12.5%	85	20.3%	1.62
Variability scenario 2	100	90	9.6%	85	15.2%	1.55
Variability scenario 3	103	90	12.1%	84	18.4%	1.50

The values in Table 2 show that the relative dose reduction following optimized decontamination is on average 51 ± 8% higher than that after normal decontamination for a fixed area of 116 m^2^ to the same distance around the house being decontaminated. This leads to the conclusion that in an authentic fallout scenario where the contamination varies, the decontamination of areas determined with isodose lines for homogeneous contamination is still a better option than decontaminating within a certain distance surrounding a building.

## Discussion

This study demonstrates the influence of the two most common building materials in Sweden, wood and brick, on the shape of the isodose lines, as well as the influence of the positions of doors and windows on the isodose lines. Including factors describing typical resident occupancy in the various rooms of the houses shows the mixture of the influence of the time spent in a specific room of the house and the continuous influence of building materials as well as positions of doors and windows. In addition, including a model for the vertical migration of contaminants in soil revealed the effects of different source depths in the soil on the decrease rate of the zones that are encompassed by the isodose lines, as well as the dominance of the contamination in the upper layer of the soil on the final isodose lines. Finally, the impact of variability in the contamination on the final result was demonstrated, and its dependence on building materials. It was shown that optimized decontamination according to isodose lines determined for homogeneous contamination is also a better choice than normal decontamination within a certain radius of the house.

In conclusion, it has now been demonstrated that the isodose concept presented in a previous study is useful for the comparison of the effects of decontaminating different surface areas, for houses constructed with different types of building materials. Downward migration of contaminants in the soil, resident occupancy, and variability in contamination were also included in the model. Further studies are required to further develop this method into a practical and useful tool for the optimization of countermeasures in cases of radioactive fallout in populated environments.

## Methods

### The isodose concept

The concept of the isodose was recently introduced by Hinrichsen *et al*.^4^, where the isodose, *ID*_*i*,*k*_, is defined by the outer boundary of one or more zones in space that contribute, for the most part, a given fraction *k* to the total dose *D*_*i*,∞_ at the observation point *i*. If ${\rho }_{D,i}(\overrightarrow{r})$ is a continuous function of the dose contribution density with the maximum *ρ*_*D*,*i*,*mac*_ < ∞, the isodose can be chosen from the range 0 < *ID*_*i*,*k*_ < *ρ*_*D*,*i*,*max*_ and the fraction *k*_*i*_ resulting from the zone or zones determined by the isodose is given by:

1 $${k}_{i}=\int \,f({\rho }_{D,i}(\overrightarrow{r}))dV/{D}_{i,\infty }\,FOR\,f({\rho }_{D,i}(\overrightarrow{r}))=\{\begin{array}{ll}{\rho }_{D,i}(\overrightarrow{r}), & {\rho }_{D,i}(\overrightarrow{r})\ge I{D}_{i,k}\\ 0, & {\rho }_{D,i}(\overrightarrow{r}) < I{D}_{i,k}\end{array}$$

The concept can also be applied to more than one observation point^4^ by introducing so-called occupancy factors, *p*_*i*_, for the various observation points into Equation 1 leading to:

2 $$\begin{array}{rcl}k & = & \int \,f({\rho }_{D}(\overrightarrow{r}))dV/\sum _{i}\,{D}_{i,\infty }\cdot {p}_{i}\,\forall \,1=\sum _{i}\,{p}_{i}\,FOR\,f({\rho }_{D}(\overrightarrow{r}))\\  & = & \{\begin{array}{ll}\sum _{i}{\rho }_{D,i}(\overrightarrow{r})\cdot {p}_{i}, & \sum _{i}\,{\rho }_{D,i}(\overrightarrow{r})\cdot {p}_{i}\ge I{D}_{k}\\ 0, & \sum _{i}\,{\rho }_{D,i}(\overrightarrow{r})\cdot {p}_{i} < I{D}_{k}\end{array}\end{array}$$

### Vertical migration of contaminants in soil

The vertical transport of radioactive contaminants in soil can be described as a function of time and vertical soil depth by a convection–dispersion model, as suggested by Schuller *et al*.^13^, Bunzl *et al*.^14^, and Kirchner *et al*.^15^:

3 $$\begin{array}{rcl}C(x,t) & = & {C}_{0}\,\exp (\,-\,\mathrm{ln}(2)\cdot t/{T}_{1/2})\cdot \,(\frac{1}{\sqrt{\pi {D}_{s}t}}\cdot \exp (-\frac{{(x-{v}_{s}t)}^{2}}{4{D}_{s}t})-\frac{{v}_{s}}{2{D}_{s}}\\  &  & \cdot \,\exp (\frac{{v}_{s}}{{D}_{s}}x)\cdot erfc(\frac{x+{v}_{s}}{2\sqrt{{D}_{s}t}}))\end{array}$$

where *C*_0_ is the initial contaminant concentration (Bq cm^−3^), *T*_1/2_ is the physical half-life (a), *D*_*s*_ is the effective dispersion coefficient (cm^2^ a^−1^), and *v*_*s*_ is the convective velocity (cm a^−1^).

### Description of the calculations

As the applicability of the Monte Carlo transport code MCNP6^16^ in determining the reduction in exposure due to shielding inside a building has been experimentally verified in a previous study on a modular building^17^, it was applied in this study. This is also valid for employed nuclear cross-section data set ENDF/B-VII.0^18^, that enables the code to account for photon creation and loss through interaction with matter. The most relevant processes in this respect are bremsstrahlung, fluorescence, Compton scattering, photon capture, pair production and p-annihilation. The complex 3-dimensional models are defined through a combinatorial geometry technique.

**Figure 13:**
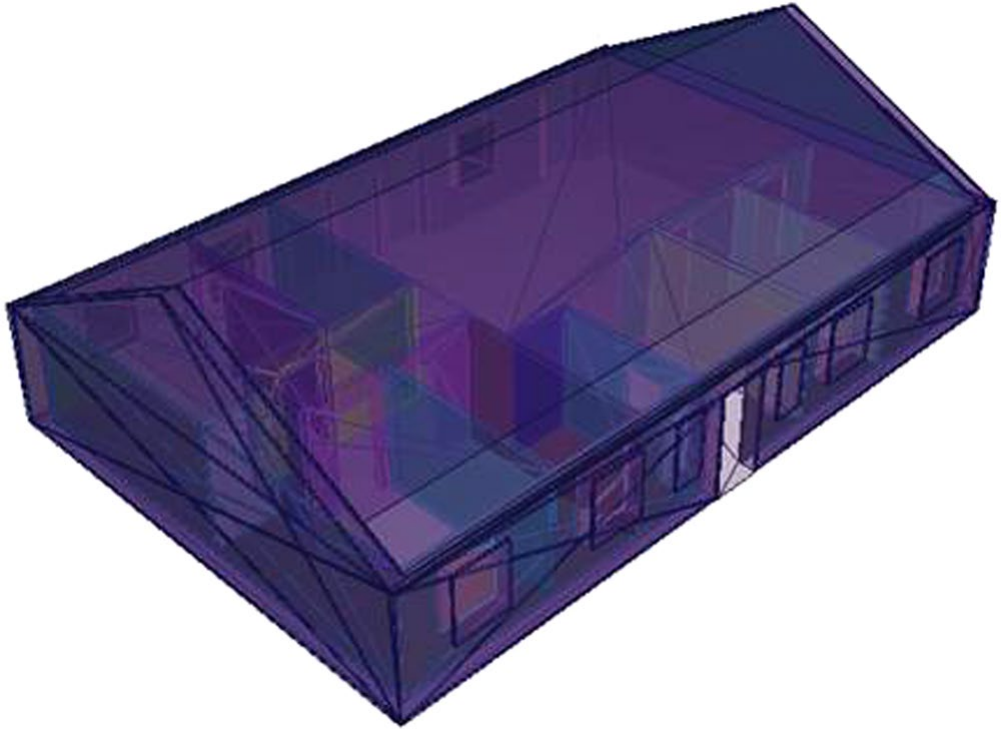
Birds-eye view of a typical Swedish house.

Two typical Swedish houses were considered, constructed with the most common building materials in Sweden, namely wood and brick. The definition of the geometries of the houses, which had similar designs, are based on the construction drawings and descriptions of actual Swedish houses made available by the Urban Planning Department of the Municipality of Hässleholm (Stadsbyggnadskontoret, Hässleholms kommun) (Fig. 13). The houses cover an area of 10 m × 15 m, and the building materials and dimensions are given in Table 3. The protection of wooden frames was assumed to be negligible and thus they are not included in the calculations. The windows and exterior doors comprise an area of 25.3 m^2^ of the total vertical area.

**Table 3:** Construction materials and dimensions for typical Swedish wooden and brick houses.

	Wooden house	Brick house
External walls	2.2 cm wood, 4 cm air, 0.9 cm gypsum, 26 cm mineral wool, 2.8 cm air, 1.1 cm wood, 1.3 cm gypsum	12 cm brick, 4 cm air, 0.9 cm gypsum, 23 cm mineral wool, 1.3 cm gypsum
Inner walls	12 cm or 17 cm gypsum	
Roof	5.4 cm concrete, 2.7 cm wood	5.4 cm concrete, 3.5 cm wood
Ceiling	1.3 cm gypsum, 2.8 cm air, 40 cm mineral wool	
Windows and doors	0.8 cm glass	

The regions in space were constructed by logical combinations (union, intersection, difference) of elementary geometric bodies and surfaces. Data from a material compendium^19^ were used to assign atomic compositions and densities to the materials, as summarized in Table 4. These were used as the input for the different building structures and environmental regions.

**Table 4:** Material specifications in terms of atomic compositions (rounded) and densities used in the Monte Carlo calculations based on the data published in a material compendium^19^.

Material	Atomic composition	Density (kg/l)
Air	0.02% C; 78.44% N; 21.07% O; 0.47% Ar	0.001205
Brick	66.34% O; 0.37% Al; 32.32% Si; 0.71% Ca; 0.25% Fe	1.8
Concrete	8.47% H; 60.41% O; 1.25% Na; 2.48% Al; 24.19% Si; 2.72% Ca; 0.47% Fe	2.25
Glass	60.39% O; 8.81% Na; 25.18% Si; 5.62% Ca	2.4
Gypsum	33.33% H; 50.00% O; 8.33% S; 8.33% Ca	2.32
Mineral wool	42.50% O; 1.70% Na; 5.40% Mg; 10.60% Al; 18.20% Si; 1.90% K; 14.30% Ca; 0.50% Mn; 4.90% Fe	0.1666667
Soil	31.69% H; 50.16% O; 4.00% Al; 14.16% Si	1.52
Wood	46.24% H; 32.34% C; 0.28% N; 20.88% O; 0.06% Mg; 0.12% S; 0.04% K; 0.04% Ca	0.64

A radioactive source energy of 0.662 MeV was assumed in the calculations as this is the energy of the gamma-rays emitted by ^137^Cs, which is the radionuclide of greatest concern in connection with the Chernobyl and Fukushima nuclear power plant accidents. Source regions were defined as 1 m × 1 m plane squares in a 1 m × 1 m grid up to a lateral distance of 10 m from the sides of the houses, at ground level, and 2.5 cm and 5 cm below ground level. Separate Monte Carlo computations were performed to obtain reference values for an infinite horizontal plane source at ground level, and 2.5 cm and 5 cm below ground level.

**Figure 14:**
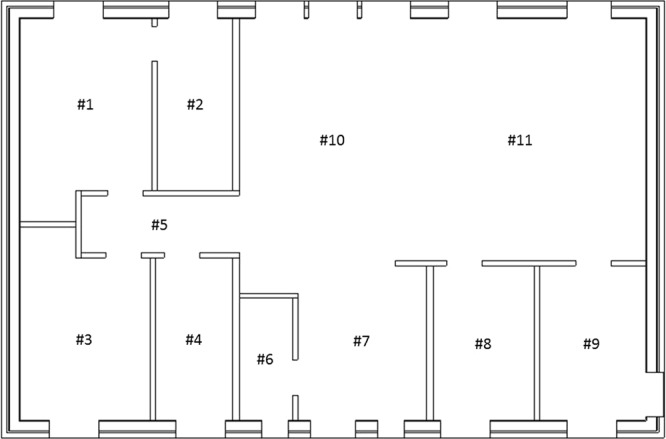
Observation points inside a typical Swedish house. #1 indicates a bedroom, #2 the bathroom, #3 a second bedroom (for example, for a child or guests), #4 a dressing room, #5 the corridor, #6 a restroom, #7 the hall, #8 a study, #9 the kitchen, #10 the living room, and #11 the dining room.

The detector regions were defined as air-filled spheres with a diameter of 30 cm, positioned 1 m above ground level, at the observation points defined in different parts of the house (Fig. 14). Observation point #1 represents a bedroom, #2 the bathroom, #3 a second bedroom (for example, for a child or guests), #4 a dressing room, #5 the corridor, #6 a restroom, #7 the hall, #8 a study, #9 the kitchen, #10 the living room, and #11 the dining room. The number and energies of the gamma ‘particles’ passing through these detector were determined with the Monte Carlo code. The fluence was transformed into air kerma free-in-air using conversion coefficients^20^.

The Monte Carlo method obtains results by averaging over the scoring of all ‘particles’ drawn by the source. Thus, the result is accompanied by a statistical uncertainty. To reach an acceptable standard deviation of below 5% within acceptable computation times MCNP6 offers various variation reduction techniques. One of them is the generation of ‘weight windows’ in connection with the cells, which are defined regions in space in MCNP6. Those weight windows make use of the weight of one MCNP particle, which gives the number of how many physical particles or photons like in this study it represents. Those can be the result of an emission from the source or an interaction of a particle with matter. Weight windows define the upper and lower limits of weights for particles entering the respective cell. The lower limits are defined by the user and the upper are calculated by a multiple of the lower limit for each cell. In case of a particle entering a cell with a lower weight than the lower weight limit of the cell, a “Russian roulette” technique is applied with the result that the particle’ s weight is either increased to a value within the limits of the respective weight window, or the particle is terminated. In case of a particle entering a cell with a higher weight than the upper weight limit of the cell, it is divided so that the resulting weights of the particles are within the limits of the weight window. In case of a particle entering a cell with a weight within the weight limits of the cell, no further actions take place. To determine the limits of the weight windows MCNP6 includes a Weight Window Generator as a tool for estimation of importance of cell in space with respect to source and detector position. The definition of the importance of a cell is the ratio of the total score because of particles and their progeny entering the cell per total weight entering the cell. Thus, by applying the cell-based generator the average importance of the cells can be estimated.

## Data Availability

The datasets generated during and analyzed during the current study are available from the corresponding author on reasonable request.
